# Correspondence

**Published:** 1912-03

**Authors:** Wm. Stanton

**Affiliations:** Univ. of Buffalo


Feb. 9, 1912.

*Editor* Buffalo Medical Journal:

Dear Doctor:

In the February Journal, page 412 I notice your comment that "it is doubtful if any Sons and Daughters (of the Revolution) in the literal sense survive."

I beg to say that Samantha Stanton Nellis of Naples, N. Y., is a real Daughter of the Revolution and still living, having celebrated her 102nd birthday January 5th. Her father Elijah Stanton was born in 1754 and the Stanton family and the Washingtons being close friends, the boy Elijah enlisted as the boy servant of Gen. Washington with whom he served until the close of the war. In 1791 he married Lucy Goodell and their home was in Fairfield, Herkimer Co., where their ten children were all born. Among these ten were Daniel, my grandfather, born April 13, 1807 and Samantha born January 5, 1810. Elijah died at the age of 103. Although I am not prepared to give you other names, I have reason to believe that a considerable number of real Daughters of the Revolution still survive.

Very truly yours,

WM. STANTON.

Univ. of Buffalo, 1889.

WE are glad to be corrected. Mrs. Sarah Bishop Carl of Pekin, Niagara Co., aged 88, has recently been mentioned in the Buffalo Express as a real D. A. R. The moral is that, sometimes, it is better not to reply on "authority" but on one's own observation. For example, the writer's great grandfather, born 1760, had a child by a second wife in 1840. This child died before its father but among the large number of boy soldiers of the Revolution, themselves born in 1760 or a few years later, quite a number must have had children at ages between 60 and 80, that is, between the years 1820 and 1845. Of these there should still be a goodly number of survivors.

